# Dataset on bio-stimulation experiments for the removal of hydrocarbons and the monitoring of certain elements in a contaminated soil

**DOI:** 10.1016/j.dib.2022.108487

**Published:** 2022-07-25

**Authors:** Simone Cavazzoli, Ville Selonen, Anna-Lea Rantalainen, Aki Sinkkonen, Martin Romantschuk, Andrea Squartini

**Affiliations:** a Department of Civil, Environmental and Mechanical Engineering, University of Trento, via Mesiano 77, 38123, Trento, Italy; b Faculty of Biological and Environmental Sciences, Ecosystems and Environment Research Programme, University of Helsinki, Niemenkatu 73, Lahti FI-15140, Finland,; c Natural Resources Institute Finland Luke, Itäinen Pitkäkatu 4 A, Turku 20520, Finland; d Department of Agronomy, Food, Natural Resources, Animals and Environment, University of Padova, Viale dell'Università, 16 - 35020 Legnaro, Veneto, Italy (PD)

**Keywords:** Soil remediation, Biodegradation, Pollutants fate and transport, Heavy metals, Bioavailability, Environmental contamination

## Abstract

Meat and Bone Meal (MBM) and β-cyclodextrin were added to a soil sample co-contaminated by hydrocarbons (diesel fraction C_10_-C_21_ and lubricant oil fraction C_22_-C_40_) and heavy metals to promote soil remediation. The pilot study was conducted in the laboratory, maintaining optimal conditions (i.e., temperature, pH, water content, soil aeration) to facilitate hydrocarbon biodegradation. Two different experimental tests were prepared: one for the analysis of hydrocarbons in soil, the other to monitor the dynamics of some elements of interest. For the first test, the two hydrocarbon fractions in the soil were quantified separately by GC-FID, following the ISO 16703:2004(E) standard protocol. Sampling and analysis were done every two weeks, for three consecutive months. For the second test (dynamics of certain elements in the soil), soil and leachate samples were analyzed by ICP-MS after appropriate pretreatment steps.

## Specifications Table

**Table:** 

Subject	Environmental Science, Pollution
Specific subject area	Hydrocarbon and heavy metal co-contaminated soil remediation. Nature-based materials as a possible solution for environmental pollution.
Type of data	Table Image Figure
How the data were acquired	ISO 16703:2004(E) standard protocol via Gas chromatography coupled with flame ionization detection (GC-FID). 6890N Network GC System Agilent Technology. Zebron ZB-5HT InfernoTM capillary column (length 15 m, inner diameter 320 µm and phase thickness 0.1 µm). Flame ionization detector (GC-FID, Agilent 6890N). Air generator connected with GC-FID: Zero Airgenerator mzAGC1L Labgas Instrument Company. GC oven temperature was started from 50 °C (hold 2 min), increased at rate 20 °C/min to 320 °C and hold for 10 min. The injection port and detector temperature were 320 and 340 °C, respectively. Hydrogen gas flow rate was set at 35 mL/min and air flow at 350 mL/min. Helium makeup gas was delivered at a rate of 25 mL/min. Inductively coupled plasma mass spectrometry (ICP-MS) analysis with Elan 6000 ICP-MS, Perkin Elmer SCIEX. Mineralization of the solid matter through MW-assisted acid digestion with MARS 6 One Touch Technology CEM Corporation equipped with PTFA containers, turntable, and optical thermometer. Temperature was hold for 20 min at 200 °C. Microscope, VWR BI500, LEICA S6E, highlight 2001 Olympus Europe, to control possible transformations in the soils
Data format	Raw Analyzed Filtered
Description of data collection	For each treatment, five replicates were analyzed, both for the oil test and the heavy metal test, and the final data reported consists in the mean value ± standard deviation, obtained from the five replicates analysis. Outliers were removed if inconsistent.
Data source location	• Institution: Department of Biological and Environmental Sciences of the University of Helsinki • City/Town/Region: Lahti, Päijänne Tavastia region • Country: Finland • Latitude and longitude (and GPS coordinates, if possible) for collected samples/data: 61.00602918936722, 25.652731719262203 Location: Niemenkatu 73, 15210 Lahti, Finland
Data accessibility	Repository name: EarthChem Library Data identification number: 2328 Direct URL to data: EarthChem Library - Repository | Dataset Information S. Cavazzoli, V. Selonen, A.L. Rantalainen, A. Sinkkonen, M. Romantschuk, A. Squartini, Dataset on bio-stimulation experiments for the removal of hydrocarbons and the monitoring of certain elements in a contaminated soil (2022) Version 1.0. Interdisciplinary Earth Data Alliance (IEDA). https://doi.org/10.26022/IEDA/112328. Accessed 2022-07-16
Related research article	S. Cavazzoli, V. Selonen, A.L. Rantalainen, A. Sinkkonen, M. Romantschuk, A. Squartini, Natural additives contribute to hydrocarbon and heavy metal co-contaminated soil remediation, Environmental Pollution 307 (2022). https://doi.org/10.1016/j.envpol.2022.119569

## Value of the Data

• The experimental data and annotations reported here are useful as they complete the overview of the research work described in the associated paper [1].
• These data may be useful to researchers and technicians who intend to work on the remediation of hydrocarbon and heavy metal co-contaminated soils. The protocols for the analysis of diesel and lubricant oil fractions by GC-FID are presented, as well as the procedures for the preparation of the so called ‘heavy metals’ samples, to be subjected to ICP-MS analysis.
• The data presented here can also be useful as a term of comparison, to assess the physico-chemical characteristics (e.g., pH, water content, hydrocarbon degradation kinetics, and heavy metal release) of contaminated soils in time.

## 1 Data Description

**Fig. 1:**
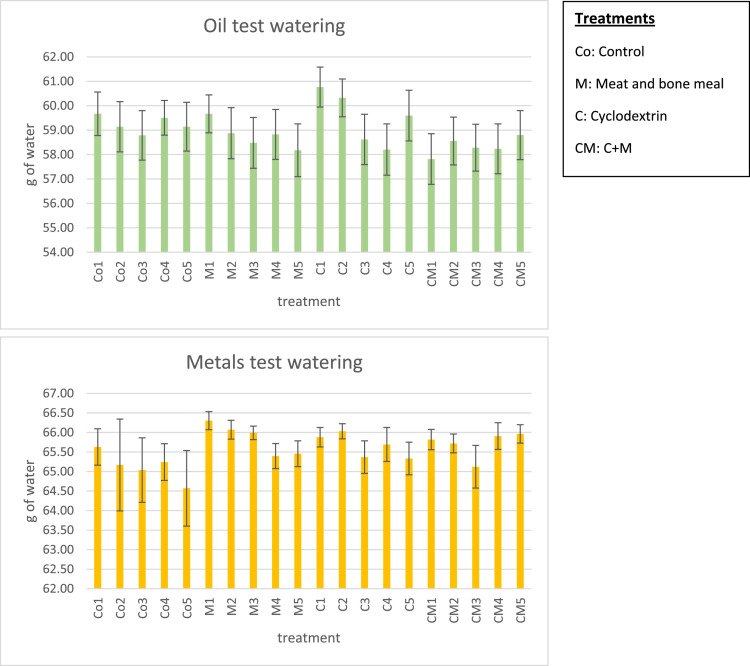
Average amount of water, in grams, contained in each treatment during the experiment, and the related fluctuations in time (±SD). The amount of water contained in each beaker was kept at around 60% of the WHC.
- Fig. 2 reports the data acquired during the determination of the water hold capacity of the as received contaminated soil. The soil was dried at 105 °C overnight, while the wet weight of the soil was noted after soaking it with tap water.

- Eurofins analysis 2018_starting soil: this is a pdf document that report the detailed analysis of the T0 soil that were commissioned to Eurofins (Eurofins Suomi - Eurofins Suomi) before the experiments started. These data were useful to state the soil contamination level.
- Fig. 1 summarizes the soil watering schedule, in both oil and heavy metal tests, during the experiment. Standard deviation represents the water content fluctuation in time. The amount of water was kept about 60% of the water hold capacity, to create the favorable condition for microbial activity.

**Fig. 2:**
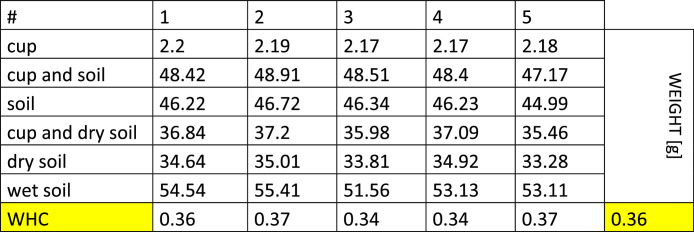
The weights of initial, dry, and wet soil after soaking for 16 h are reported. The final average value of the calculated WHC is highlighted in yellow.
- Fig. 3 reports a typical calibration curve obtained by GC-FID analysis. Two different standard stock solutions were used to prepare standard solutions with increasing concentrations (10, 50, 250, 500, 750 and 1000 µg/mL), which were then analyzed by GC-FID.

**Fig. 3:**
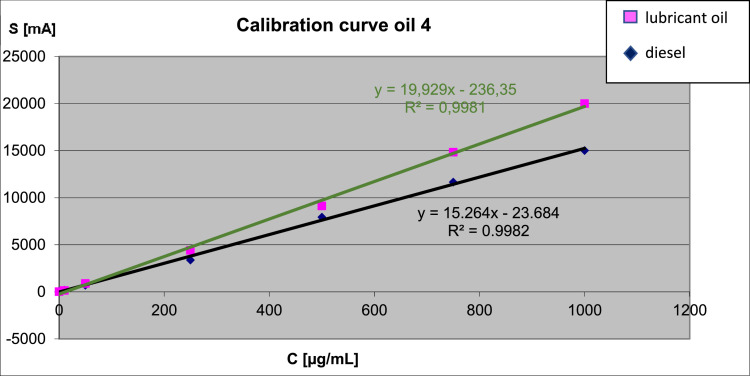
A typical calibration curve obtained from the analysis of diesel and lubricant oil standard solutions.
- Fig. 4 shows some optical microscope images, taken during the last period of the experiment (week T10 and T12). It is interesting to see the presence of mites, which appeared only in the last few weeks of the test, as well as the formation of crystalline structures, probably due to MBM and CD components added to the soil.

**Fig. 4:**
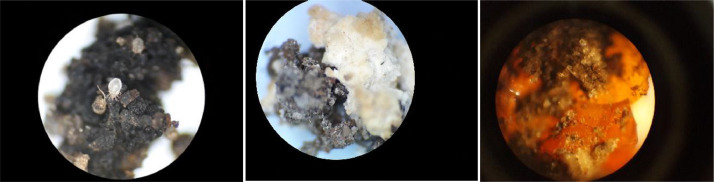
Microscope images of the treated soil. The growth of mite colonies, and crystallization phenomena were visible only in the last experimental period (8^th^ to 12^th^ weeks).
- Tables 1 and 2 collect the results (± standard deviation) obtained from GC-FID analysis of diesel and lubricant oil fractions extracted from the contaminated soil.

**Table 1:** Diesel concentrations in the various treatments. Standard Deviation (SD) is reported in brackets.

	Treatment			
t [weeks]	<Co>	<M>	<C>	<CM>
0	2880 (±207)	2880 (±207)	2880 (±207)	2880 (±207)
2	2150 (±185)	1410 (±528)	1930 (±221)	1600 (±373)
4	2200 (±768)	1390 (±366)	1850 (±534)	1140 (±348)
6	950 (±116)	720 (±169)	870 (±279)	510 (±114)
8	740 (±107)	570 (±109)	790 (±288)	430 (±140)
10	250 (±149)	230 (±47)	200 (±86)	150 (±16)
12	252 (±11)	250 (±46)	170 (±33)	120 (±26)
	
	C [mg/kg dw]

**Table 2:** Lubricant Oil concentrations in different treatments over time.

	Treatment			
t [weeks]	<Co>	<M>	<C>	<CM>
0	3820 (±506)	3820 (±506)	3820 (±506)	3820 (±506)
2	3390 (±146)	2440 (±1250)	3300 (±500)	3140 (±856)
4	5090 (±1362)	2820 (±625)	4780 (±1108)	2950 (±813)
6	2460 (±339)	1670 (±505)	2720 (±819)	1490 (±282)
8	1910 (±260)	1360 (±137)	2920 (±791)	1480 (±403)
10	610 (±292)	530 (±97)	900 (±281)	720 (±113)
12	800 (±51)	720 (±57)	960 (±149)	720 (±100)
	
	C [mg/kg dw]
- REMSOIL-MBM analysis reports the analysis carried out by Remsoil Oy (Remsoil - Innovatiivinen ratkaisu pilaantuneen maa-aineksen puhdistukseen) on the MBM, which was the natural fertilizer used as biostimulant agent in this work. Remsoil Oy is a Finnish biotechnology company, which collaborate with the University of Helsinki in soil bioremediation projects.
- Oil degradation_statistical analysis collects the analysis of variance performed with SPSS statistics software package (IBM), with MBM and CD as factors, for both diesel and lubricant oil fractions.

## 2 Experimental Design, Materials and Methods

Experimental microcosm: For both experimental tests (hydrocarbon oil removal and heavy metal mobility), 450 mL glass beakers were used to prepare four different treatments (control, Meat and bone meal, cyclodextrin and MBM/CD combination) [6], [7], [8], [9], [11], [12], five replicates each. For the HM test, polycarbonate straws were inserted in the various beakers, as to facilitate the sampling of leachate from the soil. About 100 g of granite gravel were washed with UP water and placed at the bottom of the beaker to improve aeration and make the water sampling easier. A circular piece of geomembrane (brown fabric) was placed on top of the gravels to separate the soil and rocks and to avoid mixing and packing of the soil with the rocks. Approximate weights of the materials used: glass 350 g, soil 250 g, gravel 100 g, brown fabric 0.83 g.

The frozen contaminated soil used in the experiments was thawed for seven days in the laboratory at room temperature. Subsequently, the soil was sieved and homogenized.

Watering schedule: Considering the importance of water for the microbial activity of the soil [2], [3], [4] (as well as for other chemical processes, such as hydrolysis), we kept the water content in the soil around 60% of the value of the water hold capacity (WHC, see Figs. 1 and 2). Tap water, and not deionized water, was given once a week to avoid possible damage to microorganisms. For the HM test, to obtain the leachate sample, the water load supplied exceeded the calculated WHC by approximately 20 mL, so as to obtain ∼20 mL of sample to be subjected to pH measurements and appropriate treatments for the HM analysis (see later the description). C and CM treatments (cyclodextrin and cyclodextrin + MBM) were watered with tap water + 1% (by volume) of 50% (v/v) aqueous stock solution of methyl-β-cyclodextrin [5], [13].

Sampling program: About 2 g of soil were sampled every two weeks from each beaker, transferred to a glass tube (Kimax, 15 mL) and subjected to extraction of the hydrocarbon fractions C_10_-C_21_ (diesel) and C_22_-C_40_ (lubricant oil), following the ISO 16703: 2004 (E) standard protocol explained in detail later. The sampling program for the HM test was different than the one chosen for hydrocarbon oils, as we expected that the release of the elements (i.e., heavy metals, metalloids, and phosphorus) would be rapid in the first period [10]. Thus, we sampled the leachate every week for the first three weeks, and then at week T6 and T12. The pH of the sampled solutions was systematically measured immediately after sampling. The ICP-MS quantification of the HM content in the soil was carried out at the beginning (T0), in the middle (T6) and at the end of the experiment (T12), following the method explained later in the HM analysis part. In parallel, the pH values of the soil samples were noted.

### 2.1 Basic Parameters Monitored: WHC, Humidity, OM, T, pH

For the water hold capacity calculation: $WHC=\frac{mwetsoil-mdrysoil}{mwetsoil}$ 50 g of soil (*n* = 5) was placed in a funnel with a paper filter and saturated with water by soaking them for 16 h. The soil samples were then dried overnight at 105 °C. The organic matter was obtained after placing the samples in the muffle at 550 °C for 4 h (Loss on Ignition).

The temperature was monitored with a thermometer placed in the laboratory.

The soil pH values of both tests (hydrocarbons and HM) were measured three times, i.e., at T0, T6, T12, while the pH of the percolated solutions (HM test) was measured at each sampling.

Soil pH analysis was measured according to ISO 10390: 2005 standard method, which provides for the transfer of 10 g of soil +50 mL of UltraPure (UP) water into a 100 mL flask. The solution is mixed for one hour and left to settle down for another hour. It is then possible to measure the pH by means of a pH-meter. As regards the leachate coming from the HM test, of the total 20 mL of leachate obtained (for each single beaker, *n* = 20), about 5 mL were processed and analyzed by ICP-MS for the HM quantification, while the remaining 15 mL were used as such for the pH analysis.

### 2.2 Hydrocarbon Oils

The extraction and purification procedure of the diesel and lubricant oil fraction contained in the soil samples, and the consequent analytical quantification is based on the ISO 16703: 2004 (E) standard: "Soil quality - Determination of the hydrocarbon content in the range C10-C40 by gas chromatography ". The method leads to the quantification of the petroleum hydrocarbon content in wet field soil samples by gas chromatography. It is applicable if the content of hydrocarbon oils in the soil is between 100 mg/kg and 10000 mg/kg of soil (dry weight). From the detailed chemical analyzes produced by Eurofins (see dataset, pdf), our soil contained up to 9000 mg/kg of total hydrocarbons, an amount within the limits of the method. To summarize: to prepare the retention time window (RTW) standard solution, 15 µL of n-nonane and 15 mg of n-tetracontane were added in 500 mL of n-hexane, and sonicated for 30 min. This solution, in addition to being used for the definition of the range of C_10_-C_40_ hydrocarbons, is also used for the hydrocarbon extraction from the soil, as well as for any dilutions. Increasing concentration standards were then prepared, using a concentrated stock solution of diesel and lubricant oil (10000 µg/mL).

Extraction and cleaning: 2 g of soil samples were transferred to a glass tube (∼11 g) and 4 mL of acetone were added. After gentle hand shacking, 2 mL of RTW solution were added. The tubes were then closed and shacked for one hour by mechanical stirrer at 200 rpm. The supernatant was then transferred in a clean test tube, and 2.5 mL of UP water were added. The tubes were shaken by hand thoroughly for 5 min. Using a Pasteur pipette, the polar phase, mainly consisting in water and acetone, was discarded, while the organic phase was collected in a clean test tube. In order to better remove the water from the solution, about 1/4 of a teaspoon of previously activated (160 °C for 16 h) sodium sulphate was added. To further purify the organic extract, a cleaning column was prepared by filling Pasteur pipettes with 0.5 g of activated Florisil ® (160 °C, 16 h), carefully placing a small amount of cotton at the bottom of the column (Pasteur pipette) to avoid any mass loss (Florisil ®). The organic solution was then flowed down the purification column, and the entire eluate was collected in a GC vial and immediately sealed. The first three extractions required a 1:10 (eluate: extraction solution) dilution of the extracts before the GC-FID analyzes, while over time the concentrations of hydrocarbon oils in the soil decreased, so a 1:5 dilution was sufficient. Chemstation software was used for the GC-FID analyzes. The peaks of the resulted chromatograms were integrated, separating the diesel fraction (C_10_ - C_21_) from that of the lubricating oil (C_22_-C_40_) according to the ISO 16703: 2004 (E) recommendations. The integration started just after the end of the n-nonane peak, while it ended just before the n-tetracontane peak. All chromatograms were visually verified for proper integration. The mineral oil content in the soil samples was calculated as follows:

$$Csoil\;=\;Cextract\;*\;\frac{V\;}{m}\;*\;f,$$

where C_soil_ is the hydrocarbon mass fraction in the soil sample [mg/Kg(dw)], C_extract_ is the hydrocabon mass concentration in the extracted solution, calculated from the calibration function [mg/L], *V* is the volume of the RTW–extraction solution [mL], *m* is the dry mass of the soil sample used for the extraction, and *f* is the dilution factor.

Analysis of HM in the leachate: Approximately 20 mL of leachate were collected using a Hanke Sass Wolf 100 mL syringe and transferred to a PP centrifuge tube. Centrifugation was performed at 1449*g for five minutes at room temperature (20 °C). 5 mL of supernatant were poured into a 10 mL NORM-JECT ® HSW PP syringe and filtered with a 0.2 µm PES syringe filter. 1 mL of filtered solution were then transferred to a new 15 mL PP tube and diluted 1:5 with UP water. 100 µL super pure concentrated nitric acid (65%) and 50 µl of internal standard solution (indium) were added, and the tube was closed and swirled to mix the solution well. The blank sample was prepared in the same way, with no leachate solution.

Analysis of heavy metals in the soil: The soil samples were subjected to microwave assisted acid digestion. In particular, 0.1 g of soil were placed in a PTFA container together with 10 mL of 65% HNO_3_. The tubes were closed and placed in the microwave. A thermocouple was inserted in a one of the tubes to detect the real temperature during the digestion process. The heating program was optimized by the laboratory for the specific treatment of soil samples (200°C, 20 min). At the end of the cooling phase, the acid solution was quantitatively transferred to a 50 mL volumetric flask previously filled with UP water. This procedure was done with care under the fume hood, as pressurized NO_x_ and acid fumes could be released. If the solution was slightly turbid, centrifugation was performed as above. 0.5 mL of the diluted acid solution were then transferred to a PP test tube, and a further 1:10 dilution with UP water was carried out, to obtain a 2% (v/v) HNO_3_ concentration required for the ICP-MS analyzes. 50 µL of indium internal standard were also added, and the tube with the final sample was shaken briefly in the vortex. Experimental blanks and the control samples (i.e., multi-element organometallic standard) were also prepared and analyzed. ICP-MS analyzes were carried out by the Almalab laboratory personnel (https://www2.helsinki.fi/en/infrastructures/environmental-laboratory).

## Ethics Statement

The manuscript adheres to ethics in publishing standards

## CRediT authorship contribution statement

**Simone Cavazzoli:** Conceptualization, Investigation, Visualization, Writing – original draft. **Ville Selonen:** Conceptualization, Investigation. **Anna-Lea Rantalainen:** Methodology, Resources. **Aki Sinkkonen:** Methodology, Formal analysis, Supervision. **Martin Romantschuk:** Resources, Writing – review & editing, Validation. **Andrea Squartini:** Formal analysis, Data curation.

## Declaration of Competing Interest

The authors declare that they have no known competing financial interests or personal relationships that could have appeared to influence the work reported in this paper.

## Data Availability

Dataset on bio-stimulation experiments for the removal of hydrocarbons and the monitoring of certain elements in a contaminated soil (Original data) (Earth/Chem).
